# Color-Variable Photodynamic Antimicrobial Wool/Acrylic Blended Fabrics

**DOI:** 10.3390/ma13184141

**Published:** 2020-09-17

**Authors:** Tingting Wang, Wangbingfei Chen, Tingting Dong, Zihao Lv, Siming Zheng, Xiuming Cao, Qufu Wei, Reza A. Ghiladi, Qingqing Wang

**Affiliations:** 1 Key Laboratory of Eco-Textiles, Ministry of Education, Jiangnan University, Wuxi 214122, China; wa_titing@163.com (T.W.); cwbfsophie@outlook.com (W.C.); dongtingting0203@163.com (T.D.); lzh17315534332@163.com (Z.L.); zhengsiming1013@163.com (S.Z.); qfwei@jiangnan.edu.cn (Q.W.); 2 Jiangsu Sunshine Group Co., Ltd., Jiangyin 214122, China; caoxium@163.com; 3 Department of Chemistry, North Carolina State University, Raleigh, NC 27695, USA

**Keywords:** antibacterial, photodynamic, apparent color, blended fabric, photosensitizer, rose bengal, cationic dyes, wool, acrylic

## Abstract

Towards the goal of developing scalable, economical and effective antimicrobial textiles to reduce infection transmission, here we prepared color-variable photodynamic materials comprised of photosensitizer (PS)-loaded wool/acrylic (W/A) blends. Wool fibers in the W/A blended fabrics were loaded with the photosensitizer rose bengal (RB), and the acrylic fibers were dyed with a variety of traditional cationic dyes (cationic yellow, cationic blue and cationic red) to broaden their color range. Investigations on the colorimetric and photodynamic properties of a series of these materials were implemented through *CIELab* evaluation, as well as photooxidation and antibacterial studies. Generally, the photodynamic efficacy of these dual-dyed fabrics was impacted by both the choice, and how much of the traditional cationic dye was employed in the dyeing of the W/A fabrics. When compared with the PS-only singly-dyed material, RB-W/A, that showed a 99.97% (3.5 log units; *p* = 0.02) reduction of *Staphylococcus aureus* under visible light illumination (λ ≥ 420 nm, 60 min), the addition of cationic dyes led to a slight decrease in the photoinactivation ability of the dual-dyed fabrics, but was still able to achieve a 99.3% inactivation of *S. aureus*. Overall, our findings demonstrate the feasibility and potential applications of low cost and color variable RB-loaded W/A blended fabrics as effective self-disinfecting textiles against pathogen transmission.

## 1. Introduction

Common textiles are susceptible to bacterial and fungal growth in warm and humid environments. In addition to their well-known threat to human health, these pathogens are associated with other negative effects on textiles: in addition to causing odor and mildew stains, the excretion of acid by bacteria will lead to both discoloration and mechanical damage [1], especially for protein-rich wool fabrics [2]. Medical textiles, in particular, highlight the need for an antibacterial finishing [3,4,5], as pathogens present on personal protective equipment (PPE), gowns, privacy curtains, or bedding can be easily transferred between the hospital environment, patients and healthcare workers, either directly or indirectly [6]. A number of antimicrobial agents have been incorporated into textiles for infection control, including antibiotics [7,8], quaternary ammonium salts [9,10], silver nanoparticles [11,12], graphene [13,14] and dendrimers [15,16]. However, these all exhibit certain limitations, such as being expensive and/or difficult to dye or finish on textiles, can lead to drug-resistant strains [17], are potentially toxic or lead to undesired side effects (rashes or allergic responses) on the wearer [1], or possess a limited range of antibacterial function (i.e., against a single class of pathogen) [18]. Hence, the development of safe, stable, inexpensive, and effective self-disinfecting functional textiles, particularly for biomedical applications, is of growing importance, especially in light of the dual threat posed by drug-resistant pathogens that contribute to hospital acquired infections and the ongoing COVID-19 pandemic [19,20].

Recently, the integration of antibacterial photodynamic inactivation (aPDI) with traditional textiles has been investigated as a pathogen agnostic preventive approach for infection control. The principle of aPDI is that a photosensitizer (PS), when activated by visible light absorption, will transfer energy to surrounding molecules or/and oxygen to generate reactive oxygen species by so called type I or/and type II photodynamic reaction mechanisms, respectively. Reactive oxygen species (ROS), including active free radicals and singlet oxygen, oxidize bacterial cells, thereby leading to cell death. ROS generation via the type II photodynamic reaction mechanism is much simpler than via the type I process, which involves electron transfer reactions to generate active free radicals [21]. Singlet oxygen (^1^O_2_), specifically, as the primary biocidal agent generated from the type II photodynamic mechanism, has been shown to be a high-energy, nonspecific oxidant that can inactivate bacteria, fungi, and viruses [22]. Such irreversible oxidative damage [23] has been shown to inactivate virtually all types of pathogens, including antibiotic-resistant strains [24,25]. Furthermore, as opposed to traditional antibiotics that target a specific pathway [26], a process from which drug-resistance often arises, the nonspecific and non-triggering oxidative damage by ROS are not likely to lead to the development of bacterial resistance [27]. Notably, these ROS are capable of damaging microorganisms without harming healthy cells in their vicinity owing to their limited diffusion (<200 nm for singlet oxygen) [28,29] and lifetime (~4 µs) [30,31], and, as such, there are no significant safety concerns with the use of photodynamic materials in contact with the human body.

Despite the promise of photodynamic materials for infection control, difficulties have arisen in translating laboratory-scale materials to commercial applications. Many of the previously studied photosensitizer (PS)-material systems employed either complex syntheses [32,33,34], non-scalable or expensive methods [35], and/or were of limited practicability [36,37,38]. Moreover, mono-colored nonwoven materials employing a single photosensitizer have dominated the field of photodynamic textiles, and have negatively impacted their commercialization potential due to the limited color palette available. To overcome these challenges, we have been developing dual dyed fabrics in which a photosensitizer is paired with a textile dye for improved color range. By using traditional textile dyeing methods, we also aim to avoid cost-prohibitive and non-scalable systems that cannot be easily translated to existing manufacturing infrastructures. Within this context, our group has prepared and investigated rose bengal-loaded wool/acrylic blended fabrics that exhibited favorable antibacterial properties under illumination [39]. Rose bengal (RB) is a halogenated fluorescein derivative that falls into the acid xanthene dye category [40], and which has been extensively studied in the field of photodynamic therapy since the 1980s [41,42]. It is a typical anionic water-soluble type II photosensitizer that strongly absorbs visible light in the 450–580 nm range [43], which endows textiles with a brilliant red color. The selection of RB in that previous study was not only due to its commercial accessibility, but also owing to its high ^1^O_2_ quantum yield [44] as well as its known ability to inactivate microorganisms [45]. In addition, RB has also been widely used as a dye in the diagnosis of eye diseases [46] and as a food colorant [47]; as such, its safety profile has been established, an advantage over custom-made photosensitizers. The immobilization of RB and cationic dyes on wool/acrylic (W/A) fabrics is based on the ionic affinity and van der Waals attractions between the dyes and fibers. Specifically, the negatively charged RB is attracted by the positively charged amine groups of the wool protein under acidic conditions, whereas the positively charged cationic dyes are attracted by the negatively charged acrylic containing about 1% of an acidic copolymer monomer [48]. In our previous study [39], we utilized a traditional cationic yellow dye, X-8GL, to broaden the color range of the RB-containing wool/acrylic blended photodynamic fabrics. However, questions remained as to whether other dyes beyond cationic yellow could be used in the preparation of color-variable photodynamic antimicrobial wool/acrylic blended fabrics and how different amounts of the cationic dyes either in the same or different color category affected both the colorimetric and photodynamic properties of the dual-dyed fabrics.

To address these questions, here we present our follow-up study investigating the immobilization of RB with a series of cationic dyes on W/A blended fabrics as next-generation antibacterial textiles. Traditional cationic dyes (cationic yellow X-8GL (CY), cationic blue X-GRL (CB), cationic red X-GTL (CR), cationic red FBL (CR′) and cationic red 5GN (CR″)) in different ratios (1% and 3%) were employed to enrich the color range of the W/A fabrics. The colorimetric properties (*CIELab* values) of the fabrics were characterized, and the photooxidation and aPDI efficiencies against *Staphylococcus aureus* were evaluated to assess the photodynamic behavior of the dual-dyed materials as a function of both the amount of cationic dye present as well as their light absorption profile (i.e., to determine if the cationic dye competes with the absorption of light by the photosensitizer, thereby limiting its photodynamic inactivation efficacy). Our findings will demonstrate that color-variable textiles prepared from dual-dyed (rose bengal/cationic dye) wool/acrylic blended fabrics are effective photodynamic materials that have potential applications as cost-effective medical textiles for infection control.

## 2. Materials and Methods

### 2.1. Materials

Wool/acrylic (W/A) plied yarns (32/2 count, plain weave, batch number WM0816371) were provided by Uster Technologies (Suzhou) Co., Ltd. (Suzhou, China). Rose bengal (RB, BR) and cationic red X-GTL (CR) were from Shanghai Vita Chemical Reagent Co., Ltd. (Shanghai, China). Cationic yellow X-8GL (CY), cationic blue X-GRL (CB), cationic red FBL (CR′) and cationic red 5GN (CR″) were obtained from Wuxi Xinguang Textile Co., Ltd. (Wuxi, China). All the cationic dyes were commercial level. *Staphylococcus aureus* ATCC-6538 (*S. aureus*) was from Shanghai Xiejiu Bio-Tech Co., Ltd. (Shanghai, China). All chemicals were used as received without further purification.

### 2.2. Dyeing of Wool/Acrylic Fabrics

Wool/acrylic (W/A) blended fabrics with a plain structure were knitted on a STOLL CMS 530 HP flat knitting machine (Stoll, Reutlingen, Germany) with a working width of 50″/127 cm. Sequentially, the fabrics were dyed with 3% o.w.f (on-weight-of-fiber) RB and 0, 1, or 3% o.w.f CY/CB/CR/CR′/CR″ respectively by a one-step method in a Datacolor AHIBA IR dyeing machine (Datacolor, Lawrenceville, NJ, USA). The dyeing condition was as follows: 2 g/L Na_2_SO_4_ as a leveling agent, 1:30 *w/v* bath ratio, pH 4.25 (sodium acetate buffer), 100 °C, 40 min. Finally, the dyed fabrics were washed twice in a soaping process (2 g/L soap flakes, 1:30 bath ratio, 90 °C, 30 min) followed by a rinsing process (deionized water, 1:30 *w/v* bath ratio, 90 °C, 12 h) for 4–5 times to remove any traces of the dyeing reagents.

To confirm that RB did not leach out of the dyed textiles, the fabrics were cut into circles with a diameter of 16 mm (~50 mg weight) and placed into a 24-well plate. To each well was added 100 μL phosphate buffered saline (PBS), and the samples were kept in the dark for 60 min prior to illumination under conditions that mimicked the aPDI studies (vida infra) for another 60 min. Subsequently, 300 μL PBS was added to each well, and their absorbance at 540 nm was recorded to confirm that RB did not leach out. Finally, these materials were dried at 30–50 °C and kept in the dark before further usage.

The prepared RB-only loaded fabrics will be referred to as RB-W/A [39], dual-dyed fabrics with 3% o.w.f cationic dyes will be referred to as RB/CY3-W/A, RB/CB3-W/A and RB/CR3-W/A, and the dual-dyed fabrics with 1% o.w.f cationic dyes will be designated as RB/CY1-W/A, RB/CB1-W/A, RB/CR1-W/A, RB/CR′1-W/A and RB/CR″1-W/A.

### 2.3. Physical Characterization

#### 2.3.1. Colorimetric Analysis

To prevent the light from showing through the fabrics, each sample was folded into four layers before performing the colorimetric analysis on a Datacolor 650 spectrophotometer (USA) equipped with a D65 light source at a viewing angle of 10° and aperture of 9 mm.

#### 2.3.2. Determination of RB Loadings

The determination of the RB loading was approached by a two-step dissolution process applied to the treated fabric. Approximately 0.2 g of dry, washed fabric was first immersed in 7 mL dimethylformamide (DMF) for 12 h to dissolve the acrylic fibers (as well as solubilizing the dyes fixed on them), and the sample was then rinsed with another 3 mL DMF. Second, after being dried, the remaining fibers were soaked in 10 mL wool-dissolving-solution (WDS: 50% o.w.f NaHSO_3_, 7.5 g/L SDS, 3% NaOH, 1:30 bath ratio, pH = 13) for 5 h at 90 °C in a water bath. All the obtained solutions were subsequently centrifuged (15,000 r/min, 8 min) and syringe filtered (0.22 μm) to remove undissolved fibers, and then their UV-visible spectra were recorded. Finally, the RB loading (*L*, in mol RB/g fabric) was calculated by summing the amount of RB from both steps of the dissolution process on the basis of the Beer−Lambert law (Figure S1) using the following Equation (1):

(1) $$L=C_{D}\times \;V_{D}+C_{WDS}\times \;V_{WDS}$$

where *L* is the amount of RB loaded on the fabric (mol/g), *C_D_* and *V_D_* are the concentration of RB in the volume of dimethylformamide used, and *C_WDS_* and *V_WDS_* are the concentration of RB in the volume of WDS applied.

### 2.4. Photodynamic Inactivation Studies

#### 2.4.1. Photo-Oxidation Analysis

Each pristine, single- or dual-dyed W/A fabric was cut into circle with a diameter of 22 mm (~95 mg weight), followed by immersion in 5 mL of 0.5 M aqueous potassium iodide (KI). Illumination was provided by a Xe lamp (500 W, λ ≥ 420 nm) with a sample distance of 12 cm, and an absorbance measurement at 352 nm was recorded every 10 min on a UV-2600 spectrophotometer (Shimadzu, Kyoto, Japan).

#### 2.4.2. Photodynamic Antibacterial Studies

Photodynamic antibacterial studies against *Staphylococcus aureus* ATCC-6538 were performed as previously described [33,34,49,50,51]. Briefly, the bacteria were first cultured to 10^8^–10^9^ colony forming units (CFU)/mL, centrifuged to remove the culture medium, and then resuspended in PBS solution at that same bacterial concentration. Two sets of identically prepared triplicate fabric samples with a diameter 16 mm each were individually placed into adjacent wells of two 24-well plates. A 100 μL aliquot of bacteria solution was added to each well followed by a 60 min dark incubation to promote bacteria–material contact. One plate was kept in the dark to serve as a non-illuminated control, and the other was illuminated for 60 min under conditions identical to those employed in the photooxidation studies. After illumination, 0.9 mL PBS was added to each well, and they stirred with a pipette for 10 s to re-suspend the bacteria, followed by 1:10 serial dilution for six times. Finally, a 10 μL aliquot from each serial dilution was plated onto square Tryptic Soy Agar (TSA) plates with six gridded columns before 24 h incubation at 37 °C. The survival rate was calculated by the colony number in the plates (the sample group versus the corresponding sample-free dark control), and the minimum detection limit was 0.01–0.001%.

## 3. Results and Discussion

### 3.1. Loading of 3% o.w.f. RB and 3% o.w.f. Cationic Dyes on W/A Blended Fabric

#### 3.1.1. Colorimetric Characteristics of RB/CY3-W/A, RB/CB3-W/A and RB/CR3-W/A

**Figure 1:**
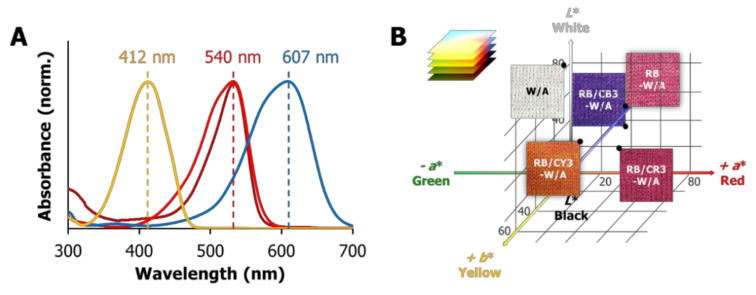
(**A**) Normalized UV-visible spectra of rose bengal (RB; maroon), cationic yellow X-8GL (CY; yellow), cationic blue X-GRL (CB; blue), and cationic red X-GTL (CR; red) in deionized water. (**B**) Photographic images of wool/acrylic (W/A), RB-W/A, RB/CY3-W/A, RB/CB3-W/A, and RB/CR3-W/A fabrics and their corresponding coordinate positions in *CIELab* color space.

To broaden the colorimetric range of the RB-loaded W/A blended fabrics, here we introduced three representative cationic dyes onto the acrylic fibers: cationic yellow (CY), blue (CB) and red (CR). As shown in Figure 1A, the maroon, yellow, blue and red curves represent the normalized UV-visible spectra of RB, CY, CB and CR dyes in deionized water, respectively. In comparison with the absorption peak of RB (~400–580 nm, λ_m__ax_ = 540 nm), that of CY (~320–470 nm) was centered at 412 nm and exhibited a minor overlap with RB, whereas the absorption of CB was centered at 607 nm and also showed a partial overlap with RB. Not surprisingly, however, CR (~400–590 nm) exhibited a nearly identical absorption profile as that of RB while sharing the same maximum absorption wavelength at 540 nm. Thus, it was expected from these solution studies that the addition of cationic dyes would influence the colorimetric characterization of RB-W/A fabric, as well as potentially their photooxidation and/or photodynamic efficacies.

The photographic images shown in Figure 1B vividly present the different colors of the loaded fabrics. The pristine W/A fabric is off-white due to the wool fibers which are rich in white protein, and it turns dark red in RB-W/A upon dyeing with rose bengal. The dual-dyed fabrics, on the other hand, exhibit overlays of the color of the dyes involved, shown as orange, purple and maroon for RB/CY3-W/A, RB/CB3-W/A and RB/CR3-W/A, respectively.

The color space coordinates in Figure 1B for the pristine W/A, RB-W/A, RB/CY3-W/A, RB/CB3-W/A and RB/CR3-W/A fabrics are provided in Table 1. The pristine W/A has the highest L* value of 81.91, with RB-W/A being half of that lightness and a much higher a* value that is in line with its reddish/pink color. The dual-dyed fabrics, as expected, were found in the red-yellow (+a*; +b*)/red-blue (+a*; -b*) and red zone (+a*) corresponding to RB/CY3, RB/CB3 and RB/CR3 loaded on W/A. Of note, RB/CR3-W/A has a much higher a* value (40.22) corresponding to the two types of red dye (CR and RB) employed when compared to the other fabrics that were loaded with only one red dye component, RB. In those cases, the a* values (29.22, 27.24 and 23.16 for RB-W/A, RB/CY3-W/A and RB/CB3-W/A, respectively) suggest a virtually identical amount of RB present, demonstrating a consistent fabric dyeing process.

#### 3.1.2. Photooxidation and Antibacterial Photodynamic Inactivation Studies of RB/CY3-W/A, RB/CB3-W/A and RB/CR3-W/A

**Figure 2:**
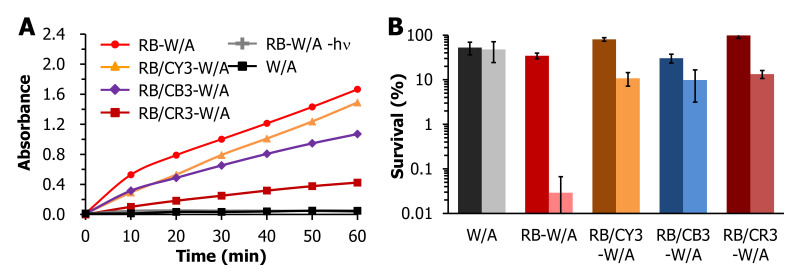
(**A**) The absorbance of I_3_^−^ (352 nm) in the 0.5 M KI solution that underwent photooxidation by RB-W/A (red), RB/CY3-W/A (orange), RB/CB3-W/A (purple), and RB/CR3-W/A (maroon) in comparison with a photosensitizer (PS)-free light control (black) and RB-W/A dark control (grey) as a function of illumination time (0–60 min). (**B**) Photodynamic antibacterial studies against *Staphylococcus aureus* using RB/CY3-W/A, RB/CY3-W/A and CY3-W/A. Displayed is the % survival for each sample in the dark (dark bar) and light (light bar) conditions. The light conditions above were as follows: 500 W, λ ≥ 420 nm, 12 cm sample distance.

Potassium iodide (KI) is a model photooxidation substrate employed to evaluate the relative production of reactive oxygen species (ROS) produced upon illumination of a photodynamic material. Specifically, iodide (I^−^) has been shown to be oxidized to I_3_^−^ by the singlet oxygen generated from RB-dyed W/A fabrics upon illumination [52]. Figure 2A depicts the absorbance value at the wavelength of 352 nm with an interval of 10 min, corresponding to the formation of I_3_^−^ in the photooxidation solution system _ENREF_43 [53]. Not surprisingly, neither illuminated W/A (PS-free light control) nor RB-W/A in the absence of illumination (dark control) formed I_3_^−^. However, the photosensitizer-loaded fabrics showed consistently increasing formation of I_3_^−^ upon visible light illumination, consistent with the production of singlet oxygen by the RB-containing W/A fabrics. Furthermore, the photooxidation ability of the fabrics followed the trend RB-W/A > RB/CY3-W/A > RB/CB3-W/A > RB/CR3-W/A, revealing that the addition of cationic dyes decreases the ROS yield of RB-W/A. This trend is consistent with the spectral overlap exhibited between RB and the cationic dyes (Figure 1A), which also followed the trend CR > CB > CY, suggesting that the dyes interfered with the absorption of light by the photosensitizer, leading to the diminished photooxidation ability of the dual-dyed fabrics in comparison with RB-W/A.

Given that the addition of the cationic dyes in the dual-dyed W/A fabrics appeared to reduce the generation of ROS, we performed aPDI studies with these fabrics to determine if their presence influenced the antibacterial photodynamic inactivation efficiencies when compared to RB-W/A. Figure 2B shows that W/A would reduce ~50% of the bacteria cells due to its fabric construction either in the presence or absence of illumination. While RB-W/A could inactivate 99.97% (3.5 log units; *p* = 0.02) of *S. aureus* under visible light illumination (60 min), RB/CY3-W/A, RB/CB3-W/A and RB/CR3-W/A were less effective, inactivating *S. aureus* by 89.2% (0.9 log units; *p* < 0.0002), 90.1% (1.0 log units; *p* = 0.02) and 86.6% (0.9 log units; *p* < 0.0006), respectively, under identical illumination conditions. The data thus demonstrated that the addition of cationic dyes at 3% o.w.f. did decrease the photodynamic antibacterial efficiency of RB-W/A, consistent with the lowered singlet oxygen production deduced from the photooxidation studies. Here, we speculate that the anionic RB and the cationic dyes may form an ion pair through electrostatic interactions, resulting in unfavorable photophysical properties. In addition, the UV-vis spectra of cationic dyes overlapped with that of RB and reduced the absorption of light by the photosensitizer, therefore decreasing the photodynamic ability of RB-W/A.

### 3.2. Loading of 3% o.w.f. RB and 1% o.w.f. Cationic Dyes on W/A Blended Fabric

In order to investigate how different amounts of the above cationic dyes (or different cationic dyes in same color category) affected both the colorimetric and photodynamic properties of the dual-dyed fabrics, we used 1% CY, CB, CR, CR′ and CR″ to further investigate their impact on the photodynamic materials. Our rationale was that a 1% loading of the cationic dyes could still effectively modulate the color of the RB-loaded fabrics without leading to the negative impacts on substrate photooxidation and/or antibacterial photodynamic inactivation that were observed with a 3% cationic dye loading.

#### 3.2.1. Colorimetric Characteristics of RB/CY1-W/A, RB/CB1-W/A, RB/CR1-W/A, RB/CR′1-W/A and RB/CR″1-W/A

**Figure 3:**
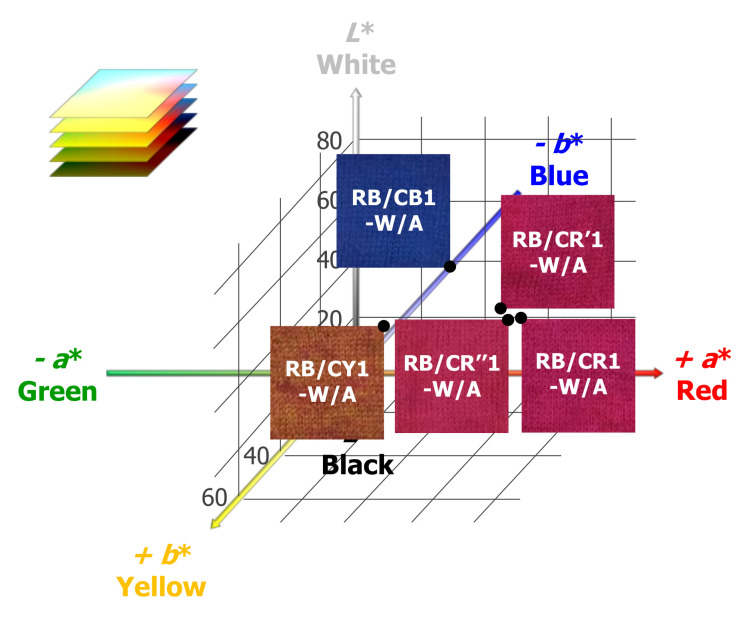
Photographic images of RB/CY1-W/A, RB/CB1-W/A, RB/CR1-W/A, RB/CR′1-W/A, RB/CR″1-W/A and their corresponding coordinate positions in *CIELab* color space.

The photographic images of the RB/CY1-W/A, RB/CB1-W/A, RB/CR1-W/A, RB/CR′1-W/A and RB/CR″1-W/A fabrics are shown in Figure 3, and they demonstrate that the 1% cationic dye loading was able to modulate the appearance of the dual-dyed fabrics in comparison with RB-W/A. With respect to the different cationic dyes in same color category (CR, CR′, and CR″), no major differences in their apparent color were observable by the naked eye for RB/CR1-W/A, RB/CR′1-W/A, RB/CR″1-W/A. All three, however, appeared to be more brightly red colored vs. RB-W/A, as shown in their corresponding CIELab values (L* = 28.92, 29.06 and 31.78, a* = 49.94, 49.92 and 54.13, b* = 7.04, 8.90 and 15.74, respectively).

As expected (Table 2), cationic yellow-dyed RB/CY1-W/A appeared to have the highest b* value (27.26), cationic blue-dyed RB/CB1-W/A presented with the most negative value of b* (−29.22), while the cationic red-dyed RB/CR1-W/A, RB/CR′1-W/A and RB/CR″1-W/A all have value a* values (49.94, 49.92 and 54.13, respectively) that were 2–3 times higher in comparison with RB/CY1-W/A (21.09) and RB/CB1-W/A (16.77). In contrast with the fabrics loaded with 3% o.w.f. cationic dyes, the 1% o.w.f. CY-loaded sample showed an obvious decrease in the absolute value of the corresponding color coordinates, as the L* and b* values dropped from 51.62 and 45.11 to the 38.55 and 27.26, respectively. The 1% o.w.f. CB and CR fabrics, on the other hand, possessed rather similar values to their 3% o.w.f. versions, which can be attributed to the absorption properties of the cationic dyes (Figure 1A) with respect to that of RB (CY overlaps the least with RB, so a variation in its loading from 1 to 3% has a much higher impact on the appearance of the dual-dyed fabric with 3% RB compared to a similar loading change with either CB or CR).

#### 3.2.2. Photooxidation and Antibacterial Photodynamic Inactivation Studies of RB/CY1-W/A, RB/CB1-W/A, RB/CR1-W/A, RB/CR′1-W/A and RB/CR″1-W/A

**Figure 4:**
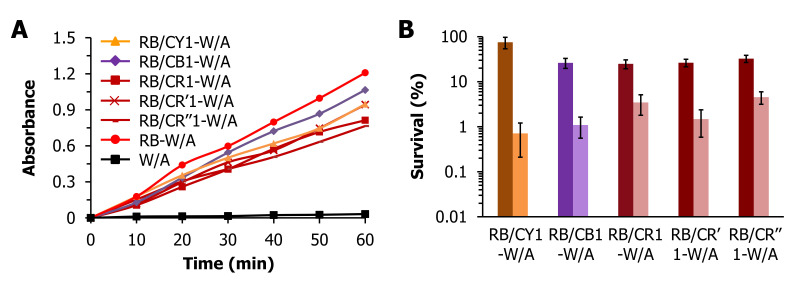
(**A**) The absorbance of I_3_^−^ (352 nm) in the 0.5 M KI solution that underwent photooxidation by RB/CY1-W/A (orange), RB/CB1-W/A (purple), RB/CR1-W/A, RB/CR′1-W/A and RB/CR″1-W/A (maroon), in comparison with a PS-free light control (black) as a function of illumination time (0–60 min). (**B**) Photodynamic antibacterial studies against *S. aureus* using RB/CY1-W/A, RB/CB1-W/A, RB/CR1-W/A, RB/CR′1-W/A and RB/CR″1-W/A. Displayed is the % survival for each sample in the dark (dark bar) and light (light bar) conditions. The light conditions were as in Figure 2.

Photooxidation studies employing KI were performed to determine if the 1% o.w.f. dye loading would attenuate the generation of singlet oxygen by RB to the same level as observed above for the 3% o.w.f. dye-loaded materials. After 60 min, the photooxidation efficacy appeared to follow the trend RB-W/A > RB/CB1-W/A > RB/CY1-W/A ≈ RB/CR‘1-W/A > RB/CR1-W/A ≈ RB/CR‘’1-W/A (Figure 4A), with this trend being generally consistent with the conclusion drawn from Figure 2A that the presence of cationic dyes in RB-loaded W/A fabrics hinders the photooxidative ability of the dual-dyed material. However, it was noted that in comparison with RB-W/A, the 1% o.w.f. cationic dyed materials led to a smaller reduction in KI photooxidation when compared to the 3% o.w.f. cationic dyed materials. This is best observed in the smaller dynamic range of the 1% o.w.f. cationic dyed fabrics (0.75–1.2 abs units at 60 min) compared with the 3% o.w.f. versions (0.3–1.7 abs units at 60 min), suggesting that while singlet oxygen production is negatively impacted by 1% o.w.f. cationic dyes, their impacts is less significant than when present at 3% o.w.f. Moreover, it is noted that the RB-W/A fabrics loaded with CR cationic dyes from the same color category (CR, CR′, and CR″) showed similar photooxidation abilities.

Compared to the photodynamic antibacterial results shown in Figure 2B, which showed ~90% (at best) inactivation of *S. aureus* by the dual-dyed RB-W/A fabrics employing a 3% o.w.f. cationic dye loading, consistently higher levels of inactivation were obtained by the 1% o.w.f. fabrics: RB/CY1-W/A (99.3%, *p* = 0.004), RB/CB1-W/A (98.9%, *p* = 0.003), RB/CR1-W/A (96.5%, *p* = 0.003), RB/CR′1-W/A (98.5%, *p* = 0.001), and RB/CR″1-W/A (95.4%, *p* = 0.001) (Figure 4B). These results are consistent with photooxidation studies above, and suggest that a smaller amount of cationic dye loading leads to improved singlet oxygen production that correlates with the observed higher levels of photodynamic inactivation. Moreover, for the three cationic red (CR, CR′, CR″)-containing materials, no statistically significant differences in the photodynamic inactivation of *S. aureus* were noted, suggesting that dyes within the same category of apparent color will likely yield the same level of inactivation in the dual-dyed fabrics.

Thus, the results here demonstrate that the photodynamic efficacy of these dual-dyed fabrics can be varied by the choice of traditional cationic dyes, and that dyes within the same color scheme (e.g., CR, CR′ and CR″) are likely to have similar results. Moreover, by avoiding overdyeing of the fabric with the traditional dye, the photodynamic antibacterial efficiency of duel-dyed materials, although less than that of the parent RB-W/A fabric, can still achieve reasonable levels of photodynamic inactivation.

#### 3.2.3. RB Loading Determination of RB/CY1-W/A and RB/CB1-W/A in Comparison with RB-W/A

To investigate whether the addition of cationic dyes on acrylic fibers would influence the loading of RB on wool, the amount of RB present in RB-W/A, RB/CY1-W/A and RB/CB1-W/A was determined by solution UV-visible spectroscopic analysis on dissolved fabric samples. The UV-visible spectra of RB in both dissolving solutions (DMF for acrylic and WDS for wool) are shown in Figure S1A,B, with characteristic absorption maximum at 562 and 548 nm, respectively. Corresponding standard curves (Figure S1C) as a function of RB concentration in the dissolving solutions allowed for the RB loading in each of following fabrics to be determined spectrophotometrically.

As can be seen from the data in Table 3, no significant differences were noted for the loading of RB on W/A as a consequence of the presence (or absence) of the cationic dye. The average RB loading of RB-W/A, RB/CY1-W/A and RB/CB1-W/A was 9.26 ± 0.19 μmol/g fabric, representing ∼31% of 3% o.w.f. RB was fixed on the W/A fabrics post-dyeing, -soaping, and -washing. We surmise that the presence of the cationic dyes does not significantly change the loading of the anionic RB on W/A blended fibers because of the strong electrostatic attraction between the cationic dye and the oppositely changed fiber. That is, cationic dye/anionic PS interactions do not appear to play a significant factor in altering the loading of RB, and likely suggests that the cationic dye/anionic fiber or anionic PS/cationic fiber electrostatics dominate, a result that was demonstrated in our previous work [39]. Given that significant differences were observed in the photodynamic properties of fabrics loaded with the same amount of photosensitizer (RB), as well as considering how a smaller ratio of the cationic dye led to better photodynamic effects, these results suggest that cationic dye loading was the key factor in inhibiting the photodynamic activity of RB, likely by absorbing light that would otherwise have led to the photodynamic production of ROS by RB.

## 4. Conclusions

Given our interest in combining aPDI technology with traditional textiles, we successfully prepared a series of variable color W/A blended fabrics using a single photosensitizer, but achieving the desired color variation through the addition of cationic dyes. In doing so, we have uncovered a competition between the absorption properties of the photosensitizer and the traditional dye in these dual-dyed materials that leads to lower photodynamic properties (e.g., photooxidation and/or bacterial inactivation) when compared to the PS-only dyed materials. However, we also note that it is possible to lessen the negative effects of this competition in one of two ways: first, by using a smaller ratio of the dye (relative to the PS), and second, by using dyes that have less absorption spectral overlap with the photosensitizer. While we employed rose bengal here in this proof-of-concept study, our results suggest that using photosensitizers and dyes with narrow absorption spectra should lead to dual-dyed materials with nearly equivalent efficacy as the PS-only dyed versions, while still achieving the desired color variation for producing textiles with a broad color palette. These findings are very promising in regard to deploying wool/acrylic blended fabrics as effective self-disinfecting medical textiles to inhibit pathogen transmission. Further investigations are currently underway to optimize this system with the goal of achieving a higher level of antibacterial activity for the commercialization of colorful traditional textiles with photodynamic properties.

## Figures and Tables

**Table 1:** *CIELab* values of pristine W/A, RB-W/A, RB/CY3-W/A, RB/CB3-W/A and RB/CR3-W/A.

Materials	L*	a*	b*
W/A [39]	81.91	−0.27	9.10
RB-W/A [39]	40.99	29.22	−13.09
RB/CY3-W/A	51.62	27.24	45.11
RB/CB3-W/A	20.39	23.16	−27.91
RB/CR3-W/A	25.86	40.22	4.03

**Table 2:** *CIELab* values of RB/CY1-W/A, RB/CB1-W/A, RB/CR1-W/A, RB/CR′1-W/A and RB/CR″1-W/A.

Materials	L*	a*	b*
RB/CY1-W/A	38.55	21.09	27.26
RB/CB1-W/A	18.85	16.77	−29.22
RB/CR1-W/A	28.92	49.94	7.04
RB/CR′1-W/A	29.06	49.92	8.90
RB/CR″1-W/A	31.78	54.13	15.74

**Table 3:** RB loadings (μmol/g fabric) of 3% o.w.f RB and 1% o.w.f CY/CB treated fabrics (RB/CY1-W/A and RB/CB1-W/A) in comparison with RB-W/A.

Dissolving Solutions	RB-W/A	RB/CY1-W/A	RB/CB1-W/A
Step 1 Dimethylformamide (DMF)	n.d.	0.46	1.13
Step 2 Wool-dissolving-solution (WDS)	9.17	8.66	8.35
Total	9.17	9.12	9.48

## Supplementary Materials

The following are available online at https://www.mdpi.com/1996-1944/13/18/4141/s1.
