# Supplementary material for: Color-Variable Photodynamic Antimicrobial Wool/Acrylic Blended Fabrics
Source: Materials (Basel). 2020 Sep 17;13(18):4141. doi: 10.3390/ma13184141 (PMC7560281; doi:10.3390/ma13184141)
Supplement: Supplementary file 1 [file materials-13-04141-s001.pdf]

Supplementary Materials

Color-Variable Photodynamic Antimicrobial Wool/Acrylic Blended Fabrics

Tingting Wang[1], Wangbingfei Chen[1], Tingting Dong[1], Zihao Lv[1], Siming Zheng[1], Xiuming Cao[2], Qufu Wei[1], Reza A. Ghiladi[1, 3*] and Qingqing Wang[1*2]

[1] Key Laboratory of Eco-Textiles, Ministry of Education, Jiangnan University, Wuxi 214122, China; wa_titing@163.com, cwbfsophie@outlook.com, 1481632893@qq.com, lzh17315534332@163.com, 1073454863@qq.com, qfwei@jiangnan.edu.cn, Reza_Ghiladi@ncsu.edu, qqwang@jiangnan.edu.cn

[2] Jiangsu Sunshine Group Co., Ltd., Jiangyin, 214122, China; caoxium@163.com, qqwang@jiangnan.edu.cn

[3] Department of Chemistry, North Carolina State University, Raleigh, North Carolina, 27695, USA; Reza_Ghiladi@ncsu.edu

* Correspondence: qqwang@jiangnan.edu.cn, (86)-1505-227-5367 (Q.W.); Reza_Ghiladi@ncsu.edu, (919)-513-0680 (R.A.G)

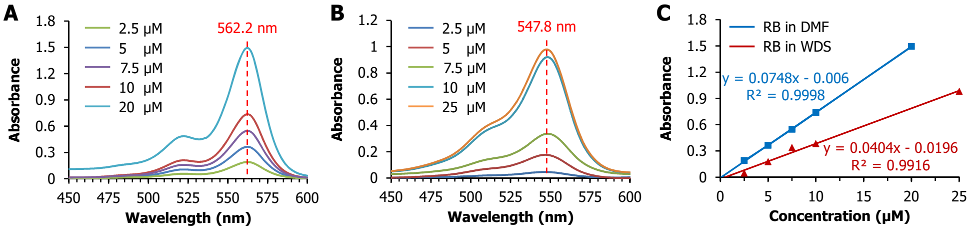

Figure S1. UV-visible spectra of RB at concentrations of 2.5, 5, 7.5, 10 and 20 μM in **A)** DMF and **B)** WDS. **C)** Standard curves of RB in DMF and WDS plotted from the data in panels A (562.2 nm) and B (547.8 nm).
